# Stathmin 1 expression in neuroendocrine and proliferating prostate cancer

**DOI:** 10.1007/s12672-025-01754-6

**Published:** 2025-01-08

**Authors:** Yingli Shi, Yunshin A. Yeh, Siyuan Cheng, Xin Gu, Shu Yang, Lin Li, Nazih P. Khater, Susan Kasper, Xiuping Yu

**Affiliations:** 1 Department of Biochemistry and Molecular Biology, Louisiana State University Health Sciences Center at Shreveport, Shreveport, LA USA; 2 Feist-Weiller Cancer Center, Louisiana State University Health Sciences Center at Shreveport, Shreveport, LA USA; 3 Pathology and Laboratory Medicine Service, Overton Brooks VA Medical Center, Shreveport, LA USA; 4 Department of Pathology, Louisiana State University Health Sciences Center at Shreveport, Shreveport, LA USA; 5 Department of Bone Marrow Transplant, Ochsner LSU Health, Shreveport, LA USA; 6 Department of Urology, Louisiana State University Health Sciences Center at Shreveport, Shreveport, LA USA; 7 Department of Environmental and Public Health Sciences, University of Cincinnati College of Medicine, Cincinnati, OH USA

## Abstract

Prostate cancer (PCa) is the second leading cause of cancer-related mortality among men in the United States. While PCa initially responds to androgen deprivation therapy, a significant portion progresses to castration-resistant PCa. Approximately 20–25% of these cases acquire aggressive neuroendocrine (NE) features, ultimately leading to neuroendocrine prostate cancer (NEPC). In this study, we investigated the expression of stathmin 1 (STMN1) across PCa subtypes using bioinformatics, western blotting, and immunohistochemical staining analyses in human and murine models. We found that elevated STMN1 expression correlated with high Gleason Scores, increased cell proliferation, and poor clinical outcomes in PCa patients. Notably, STMN1 expression was significantly higher in NEPC compared to prostate adenocarcinoma, suggesting its role in NEPC progression. Findings from TRAMP tumors, a murine NEPC model, further supported these results. In conclusion, STMN1 expression is elevated in advanced PCa, particularly in NEPC, suggesting its involvement in the progression of aggressive forms of PCa. While STMN1 shows potential as a diagnostic and prognostic marker for aggressive PCa, further studies are necessary to establish its clinical utility.

**Supplementary Information:**

The online version contains supplementary material available at 10.1007/s12672-025-01754-6.

## Introduction

Prostate cancer (PCa) is the most commonly diagnosed solid tumor and the second leading cause of cancer-related deaths among men in the United States [1]. Despite advancements in treatment modalities, including surgery, radiation therapy, and androgen deprivation therapy (ADT), the clinical management of advanced PCa remains challenging and many cases progress to castration-resistant prostate cancer (CRPC), a lethal disease state. Up to 20–25% of CRPC cases develop neuroendocrine (NE) characteristics, characterized by elevated expression of genes such as Chromogranin A (CHGA), Synaptophysin (SYP), and NCAM1 (CD56) [2]. This progression results in neuroendocrine prostate cancer (NEPC), a highly aggressive and treatment-resistant PCa subtype [3].

The TRAMP (Transgenic Adenocarcinoma of the Mouse Prostate) model is widely used to study PCa progression. In TRAMP mice, a prostate-specific probasin promoter drives the expression of SV40 T-antigen specifically in the prostate, resulting in the development of prostatic intraepithelial neoplasia (PIN), with some lesions progressing to NEPC, a process accelerated by castration [4, 5].

Stathmin1 (STMN1) is a key regulator of microtubule destabilization and plays a significant role in cell cycle regulation, migration, and differentiation [6, 7]. Elevated STMN1 expression has been linked to tumor progression and poor prognosis in multiple cancers, including lung, bladder, and liver cancers [6, 8–10]. STMN1 is also highly expressed in neurons, where it facilitates cytoskeletal remodeling, axonal growth, and synaptic plasticity [11]. While STMN1 expression has been noted in PIN and prostate adenocarcinoma (AdPC) [12], its role in aggressive PCa forms like NEPC remains poorly defined.

In this study, we evaluated STMN1 expression across PCa grades and subtypes. We found that STMN1 expression was strongly associated with high Gleason Scores, increased proliferation, and poor outcomes, particularly in NEPC.

## Materials and methods

### Bioinformatics analysis

STMN1 expression profiles and corresponding Gleason scores were retrieved from the DFKZ [13], SU2C 2019 [14] and Beltran neuroendocrine prostate cancer datasets [15] through cBioPortal. In the SU2C dataset, NEPC samples were defined by a NEPC score > 0.4 and an AR score < 0.2. STMN1 expression profiles and Gleason scores from TCGA were acquired using the R package “TCGAbiolinks”. STMN1 expression levels were transformed to log2(TPM + 1) and compared between neuroendocrine prostate cancer (NEPC) and adenocarcinoma prostate cancer (AdPC), as well as across various Gleason scores. Heatmaps showing the expression of STMN1, PCNA, TOP2A, E2F1, and AR in the SU2C and Beltran datasets were generated using the “pheatmap” package in RStudio. Transcriptomic data for STMN1 expression across various PCa cell lines were extracted from the CTPC collection [16].

To identify STMN1-associated genes, Spearman’s correlation (|r|> 0.5, p < 0.05) was performed using the SU2C RNA-seq dataset via cBioPortal. Gene ontology (GO) analysis of biological process was performed using the “clusterProfiler” package in RStudio. Survival analysis and correlation analysis between STMN1 expression and Rb mutation status were conducted using cBioPortal online tools. Patients were stratified into two groups based on STMN1 expression levels: STMN1-high and STMN1-low. Kaplan–Meier survival analysis was performed to assess overall survival. Data on STMN1 expression in normal neuroendocrine (NE) cells were extracted from the MoPSA single-cell RNA sequencing dataset collection [17], which includes samples from wild-type mice. ChIP-seq analysis of E2F1 was performed using publicly available datasets through Cistrome DB [18, 19].

### Human and murine PCa sample collection

A total of 71 human prostatic specimens were utilized in this study, obtained from Overton Brook VA medical Center, Louisiana State University Health Sciences Center at Shreveport Biorepository Core, and Tissue for Research, as described previously [20]. The specimens were classified based on histology, Gleason grades, and the expression of NEPC biomarker, insulinoma-associated-1, INSM1. These samples include benign prostate (n = 13), Gleason score 3 + 3 (n = 5), GS 3 + 4 (n = 11), GS 4 + 3 (n = 11), GS 4 + 4 (n = 8), GS 4 + 5 (n = 8), GS 5 + 4 (n = 4), GS 5 + 5 (n = 3), and NEPC tissues (n = 8). Archived TRAMP tumor sections, including both intact and castrated samples, were also used in this study. All samples were collected and utilized in accordance with protocols approved by the Institutional Animal Care and Use Committee and the Institutional Review Board of LSU Health Shreveport.

### Cell culture and western blotting analysis

PCa cell lines (VCaP, LNCaP, C42B, 22RV1, PC3, and DU145) were cultured in RPMI 1640 medium supplemented with 10% fetal bovine serum and antibiotics (penicillin and streptomycin) at 37 °C in a humidified 5% CO2 incubator. NCI-H660 cells were grown in vivo as xenograft tumors. Total proteins were extracted from PCa cells and xenograft tissues using CelLytic M cell lysis reagent (Sigma-Aldrich). Western blot analysis was performed following established protocols [20]. Antibodies against STMN1 (13655S) were obtained from Cell Signaling Technology (Beverly, MA), and antibodies against E2F1 (sc-251) and beta-actin (sc-47778) were purchased from Santa Cruz Biotechnology (Dallas, TX).

### Immunohistochemistry (IHC) and Immunofluorescence (IF) staining

Tissue sections (5-μm thick) were prepared from formalin fixed paraffin-embedded specimens, and IHC was performed following established protocols [20]. INSM1 (insulinoma-associated protein 1, sc-271408) from Santa Cruz Biotechnology (Dallas, TX) was used as primary antibody. STMN1 expression was assessed using the Allred scoring system, which combines a Proportion Score (PS) and an Intensity Score (IS) to generate a total score ranging from 0 to 8. The PS was determined based on the percentage of positively stained cells: 0 (0%), 1 (< 1%), 2 (1–10%), 3 (11–33%), 4 (34–66%), and 5 (67–100%). The IS was scored as 0 (negative), 1 + (weak), 2 + (moderate), and 3 + (strong). The total score was calculated by summing the PS and IS.

IF staining was performed according to previous protocols [20]. The primary antibodies used for IF staining included Ki67 (9449S, Cell Signaling Technology, Beverly, MA), androgen receptor (AR, sc-816, Santa Cruz Biotechnology, Dallas, TX), SV40 T antigen (TAg, sc-147, Santa Cruz Biotechnology, Dallas, TX), chromogranin A (CHGA, CPTC-CHGA-1, DSHB, Iowa City, IA), and synaptophysin (SYP, 611880, BD Biosciences).

### Statistical analysis

Group comparisons were performed using the Wilcoxon rank-sum test and the Kruskal–Wallis test, followed by post-hoc Dunn's test. Kaplan–Meier survival analysis was performed using the log-rank test. Correlations between STMN1 expression and cell proliferation markers (Ki67 & E2F1) and NEPC markers (CHGA, SYP, or NCAM1), and the Allred score of STMN1 with Gleason score were evaluated using the Spearman correlation test. A p-value of < 0.05 was considered statistically significant.

## Results

### STMN1 expression is higher in NEPC compared to AdPC

**Fig. 1:**
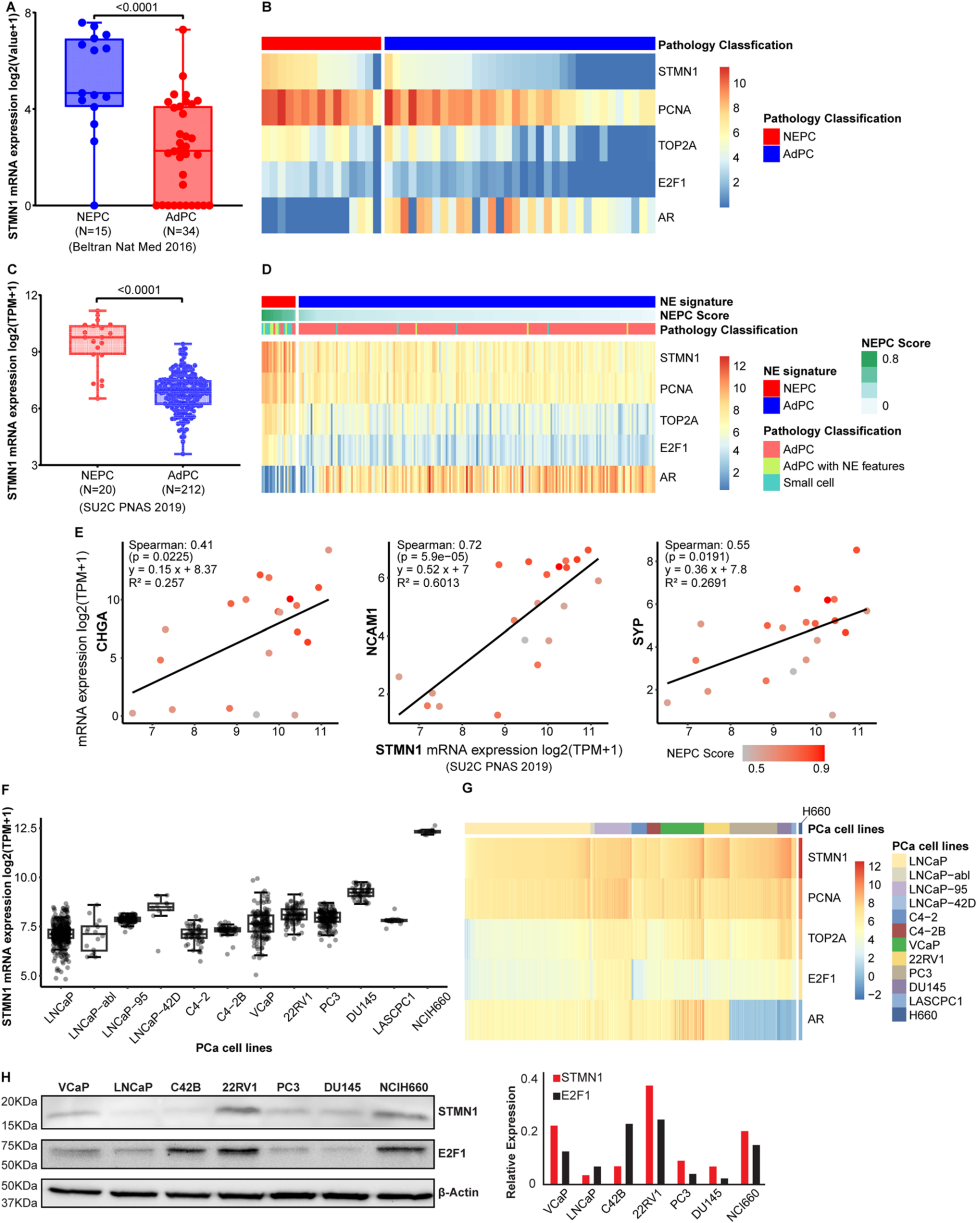
Expression profile of stmn1 in prostate cancer (PCa). The mRNA expression data of STMN1 in human PCa patients were extracted from Beltran Nat Med 2016 dataset (**A**, **B**) and SU2C PNAS 2019 study (**C**, **D**). The mRNA expression of STMN1 was significantly higher in neuroendocrine prostate cancer (NEPC) compared to adenocarcinoma prostate cancer (AdPC) (P < 0.01). Heatmaps were generated to illustrate the expression of STMN1, androgen receptor (AR) and cell proliferation markers including PCNA, TOP2A and E2F1 in NEPC versus AdPC. **E** STMN1 expression positively correlates with NEPC markers including CHGA, NCAM1 and SYP in human PCa (SU2C dataset, PNAS 2019, NEPC score > 0.4) (p < 0.02). **F**, **G** The mRNA expression data of STMN1 across various PCa cell lines were extracted from the CTPC collection. **H** Western blot analysis confirmed the protein expression of STMN1 and E2F1 in PCa cell lines, with relative quantification of western blot results shown in the right panel

STMN1 expression was analyzed using cohorts that contain both AdPC and NEPC samples, including the Neuroendocrine Prostate Cancer dataset (Beltran Nat Med 2016 [15] and SU2C 2019 [14]). The analysis revealed a significantly higher STMN1 expression in NEPC compared to AdPC (Fig. 1A, C, p < 0.001). Heatmaps showed elevated STMN1 levels in NEPC alongside proliferation markers such as PCNA and TOP2A (Fig. 1B, D). As expected, androgen receptor (AR) mRNA expression was lower in NEPC, consistent with its phenotype.

STMN1 also correlated strongly with NEPC markers (NCAM1, SYP, CHGA; Spearman’s correlations: 0.41–0.72, p < 0.02, Fig. 1E). Similarly, NEPC cell lines (NCI-H660) showed the highest STMN1 mRNA levels, accompanied by elevated proliferation markers and reduced AR expression (Fig. 1F, G). Western blot analysis confirmed these findings, showing robust STMN1 protein expression in NEPC, with variations across AdPC cell lines (Fig. 1H).

### STMN1 mRNA expression correlates with Gleason score and poor prognosis

**Fig. 2:**
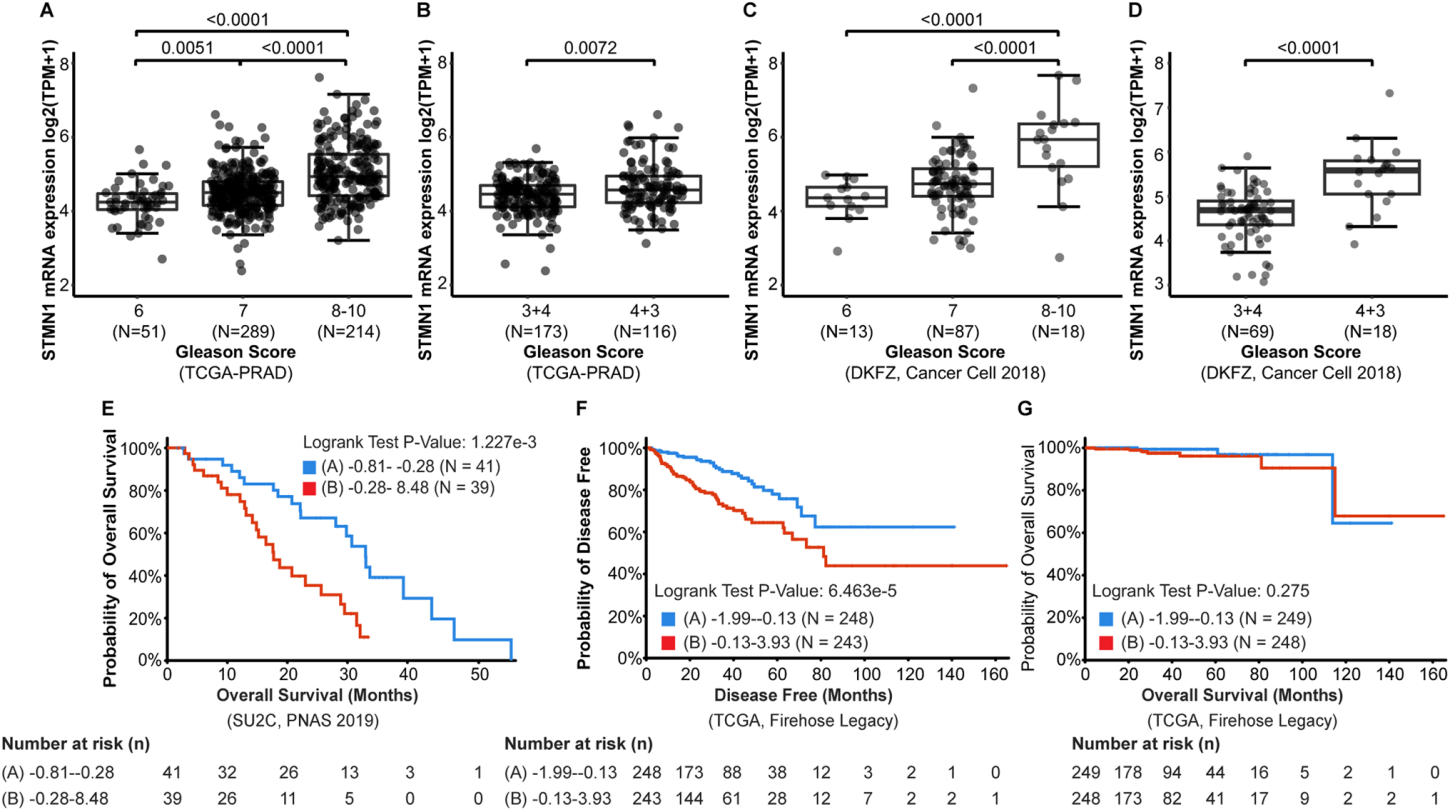
Association of STMN1 expression with tumor grade and clinical outcomes in prostate cancer (PCa). Gene expression and clinical data were extracted from the TCGA-PRAD (**A**, **B**) and DFKZ (**C**, **D**) datasets. **A**, **C** STMN1 expression levels were significantly higher in high-grade tumors (GS ≥ 8) compared to low-grade (GS 6) and intermediate-grade tumors (GS 7) in both TCGA-PRAD and DFKZ datasets. **B**, **D** STMN1 expression was significantly higher in the GS 4 + 3 group compared to the GS 3 + 4 group in both datasets. **E**, **F** Kaplan–Meier survival analysis of the SU2C (PNAS, 2019) and TCGA (Firehose Legacy) datasets showed significantly shorter overall survival and disease-free survival, respectively, in patients with high STMN1 expression compared to those with low STMN1 expression (p < 0.01). **G** No significant difference in overall survival was observed between high and low STMN1 expression groups in the TCGA (Firehose Legacy) dataset

STMN1 expression was analyzed across various grades of PCa, ranging from low to high grade, using data from the TCGA-PRAD and DKFZ datasets. This revealed increased STMN1 expression in high-grade tumors (Gleason score [GS] 8–10) compared to lower-grade tumors (p < 0.01, Fig. 2A, C). Among GS 4 + 3 and GS 3 + 4 tumors, STMN1 was higher in the more aggressive GS 4 + 3 subgroup (p < 0.01, Fig. 2B, D).

Clinical outcome analyses linked high STMN1 expression to reduced overall survival in SU2C dataset, (p < 0.01, Fig. 2E). Although there was no significant difference in overall survival rates between high and low STMN1 expression group in TCGA-PRAD dataset (p = 0.41, Fig. 2G), patients with high STMN1 expression exhibited a significantly lower disease-free survival probability (p < 0.01, Fig. 2F).

### STMN1 protein expression is elevated in NEPC and advanced AdPC

**Fig. 3:**
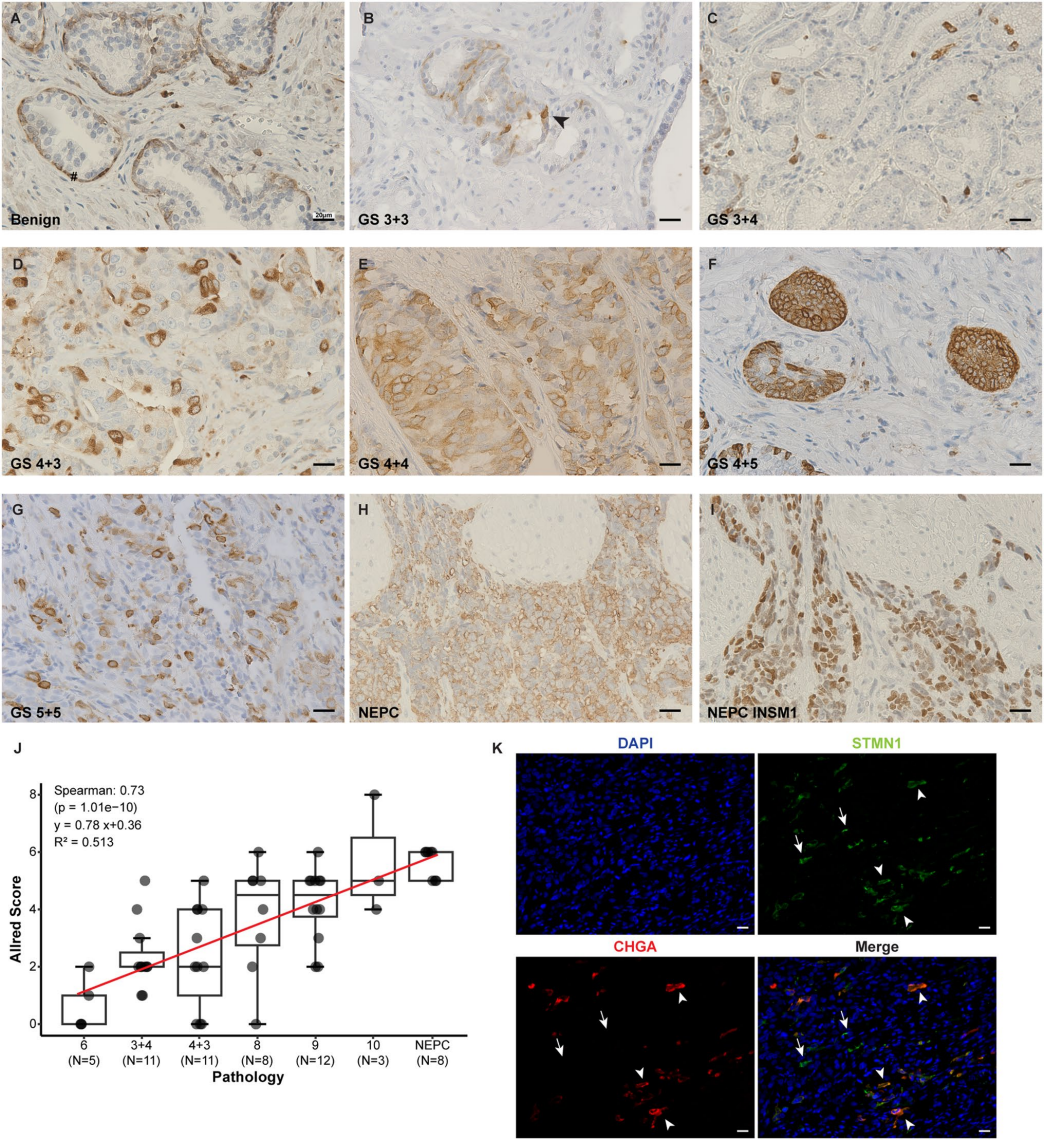
The expression of STMN1 protein in human prostatic tissues. **A**–**H** Representative immunohistochemical staining of STMN1 in human prostatic tissues. **A** Positive STMN1 staining was observed in basal epithelial cells in benign prostatic tissue. **B**–**G** Varying intensity of STMN1 staining were present in luminal and/or basal epithelial cells in adenocarcinoma tissues. The # symbol and arrowhead indicated basal epithelial cells and luminal epithelial cells, respectively. **H** and **I** Serial sections of a NEPC tumor showed STMN1 expression in the NEPC area (highlighted by the expression of NEPC marker, INSM1) but no expression was detected in adjacent AdPC cells. **J** Distribution of STMN1 expression (Allred score) among prostate specimens, demonstrating an association between STMN1 expression and Gleason Score of PCa. (Spearman correlation: 0.73, p < 0.01). **K** Dual immunofluorescence staining to access the co-expression STMN1 with NEPC marker Chromogranin A (CHGA) in AdPC tumor with NE differentiation. STMN1 expression was detected in both NE (arrowheads) and non-NE (arrows) cells. Scale bars = 20 μm

Immunohistochemical (IHC) staining revealed that STMN1 was predominantly localized to basal epithelial cells in benign tissues but absent in luminal cells (Fig. 3A, n = 13).

In cancer tissues, STMN1 expression was quantified using Allred scores, revealing a significant association with tumor grade in AdPC. STMN1 expression was undetectable or minimal (Allred score < 3) in all GS 6 and 81% of the GS 3 + 4 cases. Weak STMN1 expression (Allred score 3–4) was observed in 36.3% of GS 4 + 3 cases, while moderate to high expression (Allred Score > 4) was noted in 56% of GS 8–10 tumors (Fig. 3B–G). A strong positive correlation was observed between STMN1 expression and PCa pathology (Spearman correlation: 0.73, p < 0.01, Fig. 3J).

In NEPC, STMN1 expression was consistently high (Allred score > 4, n = 8, Fig. 3H). Serial sections stained with an anti-INSM1 antibody delineated NEPC regions, further confirming the distinct localization of STMN1 (Fig. 3I). Additionally, dual immunofluorescence staining demonstrated co-expression of STMN1 and the NE marker Chromogranin A (CHGA) in NEPC cells scattered within an AdPC tumor exhibiting NE differentiation (Fig. 3K).

These findings highlight the elevated expression of STMN1 in NEPC and advanced AdPC, suggesting its potential role in aggressive PCa progression.

### STMN1 is expressed in neuroendocrine cells in normal and benign prostate

**Fig. 4:**
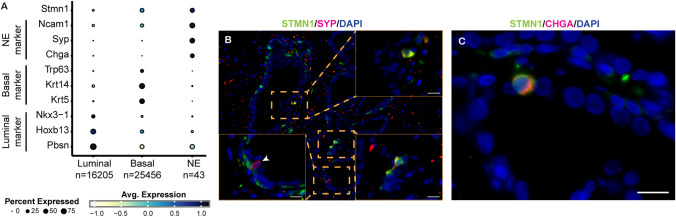
Stmn1 expression in normal neuroendocrine (NE) cells. **A** Stmn1 expression in normal NE cells in murine prostate. Single-cell RNA sequencing data from prostates of wild-type mice were obtained from Sawyers’ study collected in MoPSA [17, 21]. Specific markers used to identify different cell types include Nkx3-1, Hoxb13, and Pbsn for luminal epithelial cells; Krt5, Krt14, and Trp63 for basal epithelial cells; and Chga, Syp, and Ncam1 for NE cells. **B**, **C** Dual immunofluorescence staining showing co-expression of STMN1 with NE markers CHGA or SYP in human benign prostate specimens. STMN1 expression was detected in cells positive for SYP or CHGA expression. Arrowhead in (**B**) indicated a STMN1-negative NE cell. Scale bars = 20 μm

Investigating STMN1 expression in normal NE cells proved challenging due to the limited availability of prostate specimens from healthy individuals and the low abundance of NE cells in normal prostatic tissues. To address this, single-cell RNA sequencing (scRNA-seq) data from wild-type mice were analyzed [17, 21]. Among 85,291 cells, normal NE cells constituted approximately 0.05% of the total population. Notably, Stmn1 expression was enriched in NE cells compared to luminal or basal epithelial cells, alongside other NE markers such as Ncam1, Syp, and Chga (Fig. 4A).

To evaluate STMN1 expression in NE cells of human benign prostatic tissues, dual immunofluorescence staining was performed using antibodies against STMN1 and the NE markers Chromogranin A (CHGA) or Synaptophysin (SYP). NE cells co-expressing STMN1 with SYP and CHGA were identified (Fig. 4B, C). However, a rare NE cell exhibited no detectable STMN1 staining (arrowhead, Fig. 4B).

In summary, STMN1 expression was consistently detected in NE cells of both murine normal prostate tissues and human benign prostate tissues, suggesting its conserved role in NE cell biology.

### STMN1 expression correlates with a proliferative phenotype

**Fig. 5:**
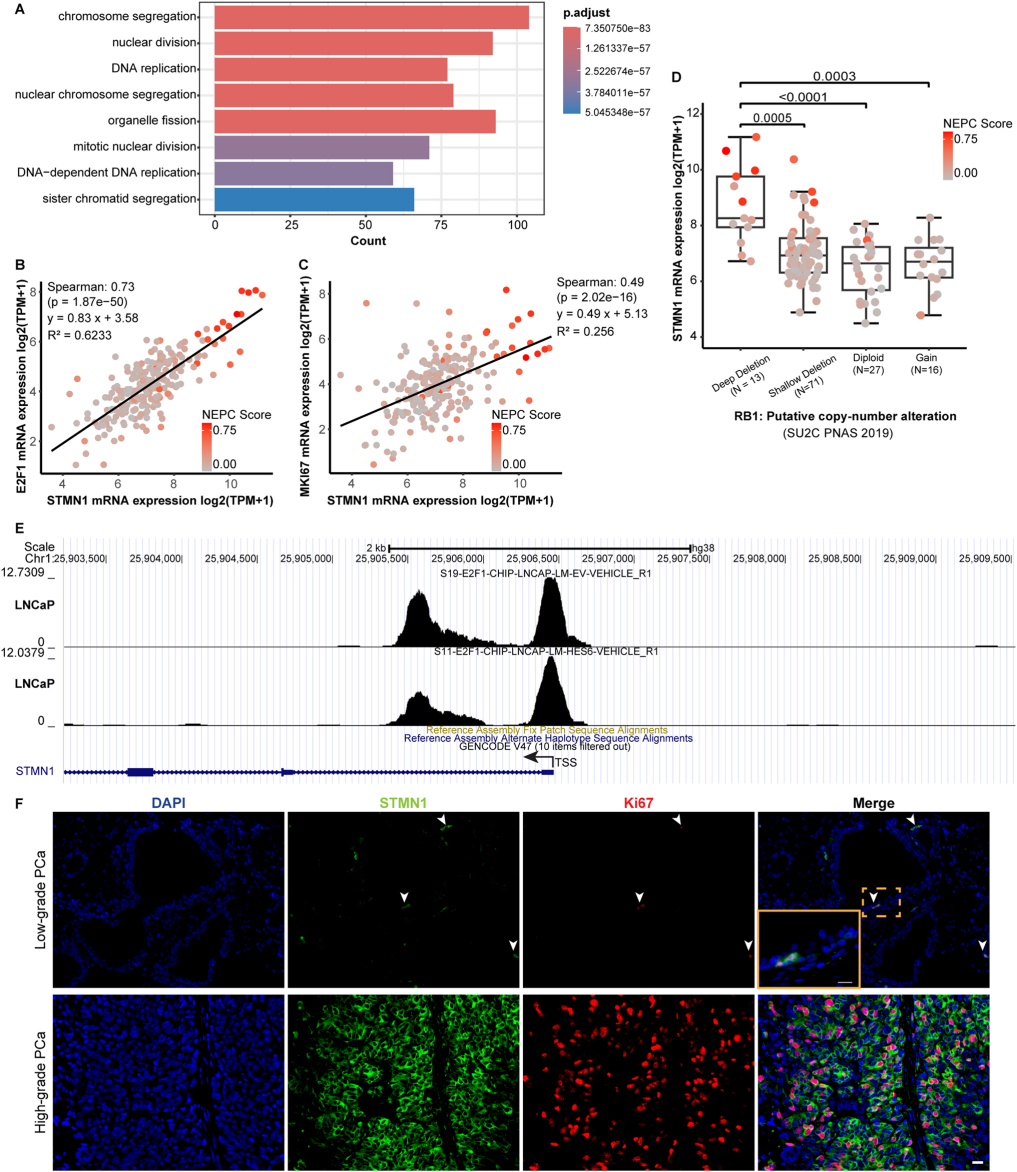
Correlation of STMN1 expression with cell proliferation in PCa. **A** Gene ontology (GO) analysis revealed pathways enriched in STMN1-positive PCa. **B**, **C** STMN1 expression positively correlates with Ki67 (**B**) and E2F1 (**C**) in human PCa (SU2C dataset, PNAS 2019) (p < 0.01). **D** STMN1 expression was significantly higher in RB1-deleted PCa group compared to the diploid group, based on data extracted from SU2C 2019 dataset. **E** E2F1 binding peaks were identified near the transcription start site of STMN1 through analysis of publicly available ChIP-seq datasets **F** Dual immunofluorescence staining to show the co-expression of STMN1 in Ki67-positive cells in AdPC. The arrowhead denoted cells co-expressed Ki67 and STMN1. Scale bar = 20 μm

To identify genes associated with STMN1 expression in PCa, an analysis of the SU2C 2019 cohort was conducted. This identified a total of 362 STMN1-correlated genes (|Spearman's correlation|> 0.5, p < 0.05). Gene Ontology (GO) analysis of these genes revealed significant enrichment in biological processes related to cell division, including “chromosome segregation,” “nuclear division,” “DNA replication,” and “mitotic nuclear division” (Fig. 5A), suggesting a potential role for STMN1 in promoting cell proliferation.

Consistent with this, significant correlations were observed between STMN1 expression and key markers of cell proliferation, such as Ki67 (p < 0.01, Fig. 5B) and the cell cycle regulator E2F1 (p < 0.01, Fig. 5C) in PCa. Analysis of publicly available ChIP-seq datasets revealed E2F1 binding sites at the STMN1 locus (Fig. 5E), indicating that E2F1 may directly regulate STMN1 transcription.

The relationship between STMN1 and RB1, a key regulator of cell proliferation through E2F1, was also explored. Analysis revealed that STMN1 mRNA expression was significantly higher in specimens with RB1 deep deletions compared to those with shallow deletions, RB1-diploid, or RB1 gain (p < 0.01, Fig. 5D).

Finally, dual immunofluorescence (IF) staining confirmed co-expression of STMN1 and Ki67 in both benign and adenocarcinoma prostate tissues (Fig. 5F), further supporting the association of STMN1 expression with a proliferative phenotype.

### Stmn1 expression in TRAMP models mirrors tumor progression

**Fig. 6:**
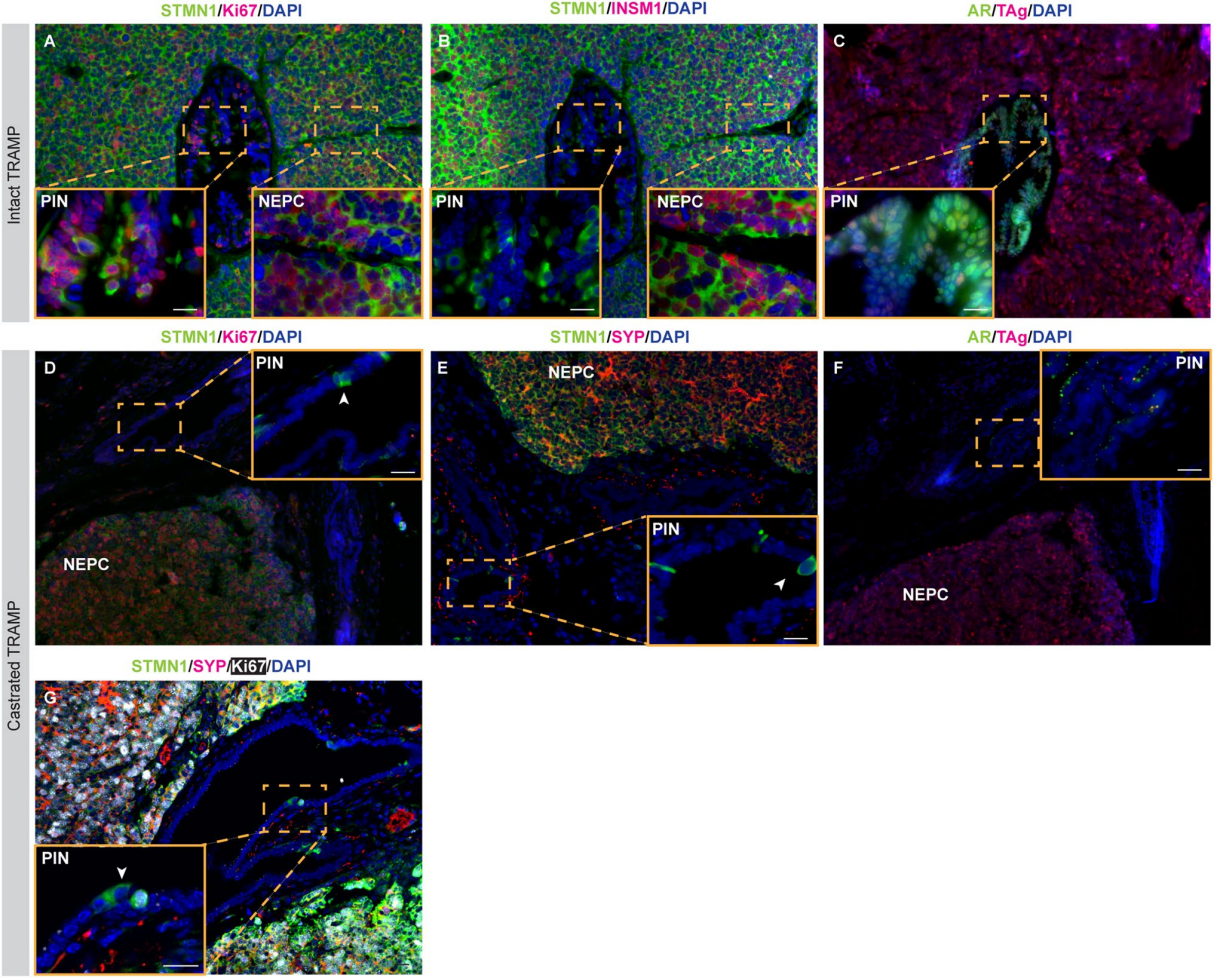
Immunofluorescence (IF) staining of STMN1 in TRAMP tumors from intact (**A**–**C**) and castrated mice (**D**–**G**). **A**–**C** In intact TRAMP tumors, high STMN1 expression was detected in both NEPC areas (approximately 100% of NEPC cells, indicated by the co-expression of INSM1) and adjacent PIN lesions (over 50% of PIN cells, which lacked INSM1 expression). While TAg was expressed in both PIN and NEPC cells, notable AR protein was detected in PIN cells but not in NEPC cells (**C**). In castrated TRAMP tumors, STMN1 expression was detected in almost all NEPC cells but was largely absent in PIN cells (**D**–**F**). Rare Stmn1-positive cells in the PIN lesions did not express Ki67 (**D**), SYP (**E**), or TAg (**F**), indicating the presence of non-proliferating, non-NE cells that express Stmn1. **G** Triple IF staining for STMN1/SYP/Ki67 revealed co-expression of STMN1 with Ki67 and SYP in NEPC. Some rare STMN1-positive cells in the PIN lesions did not express SYP nor Ki67. The arrowhead denotes a STMN1-positive cell that was neither proliferating nor NE. N > 3 for each group. Scale bar = 20 μm

In TRAMP tumors, STMN1 expression was consistently observed in NEPC cells, regardless of castration status, but was largely absent in PIN lesions from castrated mice (Fig. 6A–G). Specifically, NEPC cells exhibited high levels of STMN1, T-antigen (TAg), Ki67, and NE markers including INSM1 and SYP, while lacking AR expression. In contrast, PIN lesions in intact mice expressed STMN1, AR, TAg and Ki67. However, in castrated TRAMP mice, expressions of STMN1, Ki67, AR and Tag were markedly reduced in PIN cells (Fig. 6A–G), with only a rare subset of PIN cells (< 5%) showing STMN1 expression (Fig. 6D, E). Interestingly, some STMN1 + PIN cells lacked Ki67, TAg or NE marker SYP expression (arrowhead, Fig. 6G).

Taken together, these findings indicate that STMN1 expression is associated with cell proliferation and the NE phenotype, independent of androgen status.

### STMN1 is the predominant isoform in PCa

**Fig. 7:**
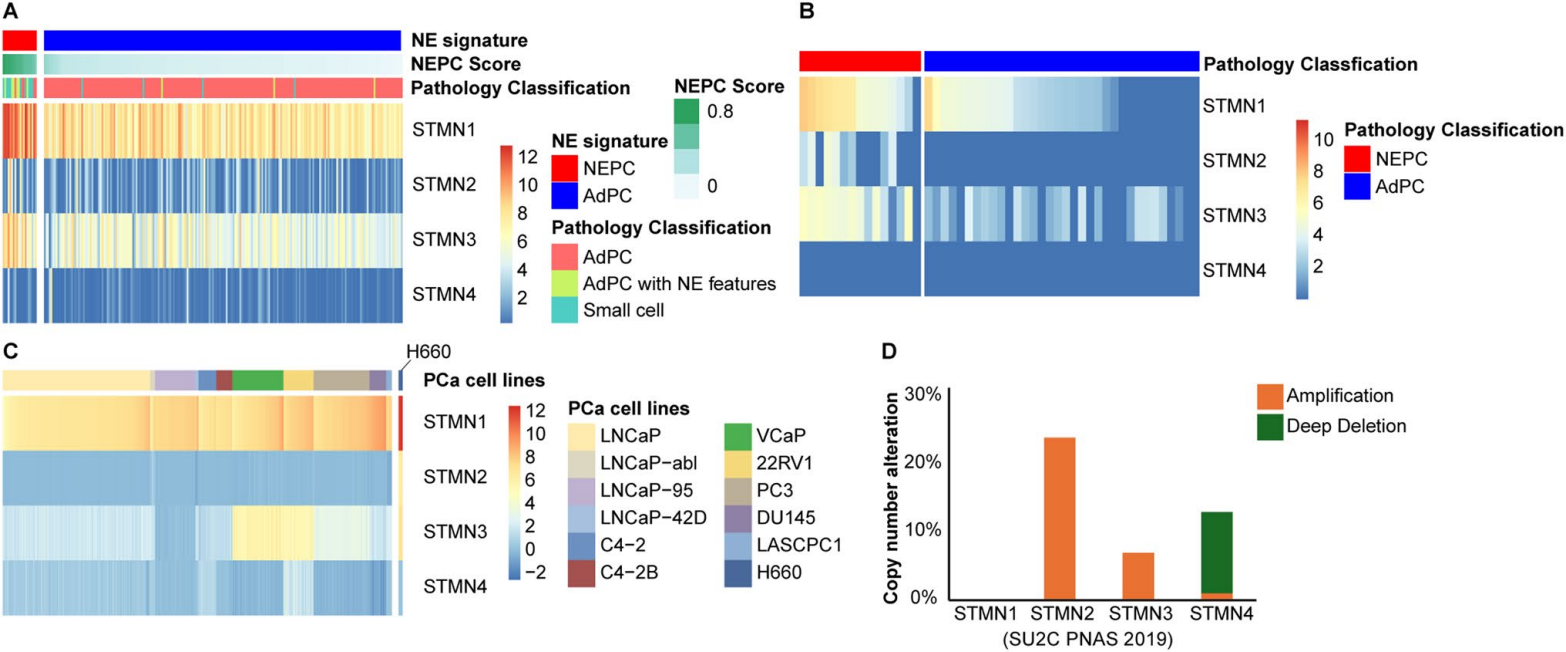
Differential expression of STMN family members in PCa. **A**, **B** Heatmap showing the expression levels of STMN family members in patient samples. STMN1 was the predominant isoform, followed by STMN3. Both STMN1 and STMN3 mRNA levels were significantly higher in NEPC compared to AdPC (p < 0.01). Gene expression data were extracted from two RNA-seq datasets SU2C (**A**) and Beltran (**B**). **C** Heatmap of STMN family members in PCa cell lines. RNA-seq data were extracted from CTPC collection. **D** Copy number alterations of STMN family members in PCa, based on SU2C cohorts

To determine the predominant STMN family member in PCa, in-silico analyses were performed using the SU2C cohort [14], Beltran cohort [15], and CTPC collection [16]. These analyses included patient samples and various PCa cell lines. As shown in Fig. 7A, B, STMN1 emerged as the most highly expressed isoform in PCa specimens, followed by STMN3, while STMN2 and STMN4 showed comparatively low expression levels. Notably, STMN3 expression was significantly higher in NEPC compared to AdPC (p < 0.01), mirroring the expression patterns observed for STMN1. High levels of STMN1 were also observed in NEPC NCIH660 cells (Fig. 7C).

Genomic analysis revealed that STMN1 had a lower rate of copy number alterations (< 1%) compared to other STMN family members in the SU2C cohort (Fig. 7D). This highlights its regulatory significance. By contrast, the STMN2 gene was amplified in 24% of cases, STMN3 in 7%, while STMN4 showed a deep deletion in 12% of samples.

## Discussion

In this study, we conducted comprehensive analysis, incorporating multiple RNAseq datasets, ChIPseq data, and IHC and IF staining of both human and murine specimens. We identified STMN1 expression in NEPC, an aggressive subtype of PCa associated with poor prognosis and limited therapeutic options. Notably, STMN1 was also expressed in normal NE cells, suggesting its potential role in maintaining the NE phenotype. Additionally, elevated STMN1 expression was detected in proliferating prostate adenocarcinoma cells, where it correlated with higher tumor grades and worse clinical outcomes. These findings suggest that STMN1 could serve as a prognostic marker for PCa, but further validation in larger patient cohorts is required to confirm its clinical utility.

A key finding of this study is the potential direct transcriptional regulation of STMN1 by E2F1 and the correlation between STMN1 expression and RB1 loss, a critical tumor suppressor in PCa. RB1 regulates cell cycle by binding to E2F transcription factors, inhibiting their transcriptional activity [22]. Loss of RB1 in advanced PCa, particularly in CRPC and NEPC, is associated with poor prognosis, with RB1 loss exceeding 70% in these subtypes [14, 23, 24]. We found that STMN1 expression was positive correlated with both RB1 loss and increased E2F1 expression, suggesting a potential crosstalk between the RB/E2F1 axis and STMN1 regulation. This was further supported by our ChIPseq analysis, which identified E2F1 binding sites in the STMN1 promoter region. Our findings are consistent with previous research showing that E2F1 can transactivate STMN1 expression in hepatocellular carcinoma [25], reinforcing the regulatory relationship between the RB/E2F1 axis and STMN1.

The TRAMP model further supports this relationship, highlighting the potential impact of RB1 inactivation on STMN1 expression. The TRAMP model is a T-antigen transgenic mouse model in which T-antigen expression is driven by an androgen-responsive probasin promoter. In intact TRAMP mice, T-antigen is expressed in both PIN and NEPC cells. However, in castrated TRAMP mice, T-antigen expression is absent in PIN cells due to inactive AR signaling but persists in NEPC cells, driven by the expression of Foxa2 [26]. In this model, the castration-induced changes in T-antigen expression parallel the expression patterns of STMN1, suggesting a mechanistic link between RB1 inactivation and STMN1 regulation.

Interestingly, in the PIN lesions of castrated TRAMP mice, we identified rare STMN1-positive cells that were negative for T-antigen, Ki67 and Synaptophysin (Fig. 6G). This suggests a potentially novel role for STMN1-expressing cells in PCa progression, particularly in the context of the NE differentiation. These Stmn1-positive cells, which lack the expression of T-antigen, proliferation markers, and NE markers, may represent a transitional population moving from adenocarcinoma to NEPC, a process previously observed during PCa progression [3, 27]. The presence of STMN1 in these cells could reflect cytoskeletal remodeling and cell differentiation, key features of the transition to more aggressive and invasive cancer phenotypes. Further studies, such as lineage tracing, single-cell RNA sequencing, or the identification of additional molecular markers, are needed to better define the identity of these cells and elucidate their precise role in the adenocarcinoma-to-NE transition in PCa.

STMN1 plays a crucial role in microtubule dynamics, which are essential for both mitotic entry and exit, and has been implicated in chemotherapy resistance [8, 28–31]. Taxane chemotherapy, which targets microtubules [32], can be less effective when STMN1 levels are elevated, as STMN1 mediates resistance by de-stabilizing microtubules. In non-small cell lung cancer (NSCLC) and small cell lung cancer (SCLC), high STMN1 expression correlates with poor chemotherapy response and shorter survival [33–38]. Similarly, the elevated expression of STMN1 in advanced PCa raises concerns about the effectiveness of chemotherapy in these patients. Our findings suggest that STMN1 could serve as a clinical indicator of poor response to taxane-based treatments, and that targeting STMN1 may help overcome taxane resistance, potentially improving treatment outcomes in advanced PCa.

In conclusion, our study presents compelling evidence that STMN1 is highly expressed in aggressive PCa, particularly NEPC, and is associated with increased proliferation and poorer survival. These findings position STMN1 as a promising candidate for further investigation as both a biomarker and a therapeutic target in PCa, particularly for overcoming chemotherapy resistance and improving treatment outcomes.

## Supplementary Information

Supplementary Material 1: Supplementary Figure 1. Original western blot images showing protein expression of STMN1 (A) and E2F1 (B) in prostate cancer (PCa) cell lines, with β-actin (C) used as a loading control. The cell lines, arranged from left to right, are VCaP, LNCaP, C42B, 22RV1, PC3, DU145, and NCI-H660.

## Data Availability

All public data listed in the “Methods” section can be assessed through cBioportal (https://www.cbioportal.org/), Cistrome Data Browser (http://cistrome.org/db/#/), CTPC (https://pcatools.shinyapps.io/CTPC_dev/) and HuPSA-MoPSA (https://pcatools.shinyapps.io/HuPSA-MoPSA/). Data sharing is not applicable to this article as no datasets were generated during the current study.
