# Supplementary material for: Stathmin 1 expression in neuroendocrine and proliferating prostate cancer
Source: Discov Oncol. 2025 Jan 8;16:19. doi: 10.1007/s12672-025-01754-6 (PMC11711591; doi:10.1007/s12672-025-01754-6)
Supplement: Supplementary file 1 — Supplementary Material 1: Supplementary Figure 1. Original western blot images showing protein expression of STMN1 (A) and E2F1 (B) in prostate cancer (PCa) cell lines, with β-actin (C) used as a loading control. The cell lines, arranged from left to right, are VCaP, LNCaP, C42B, 22RV1, PC3, DU145, and NCI-H660. [file 12672_2025_1754_MOESM1_ESM.pptx]

## Slide 1
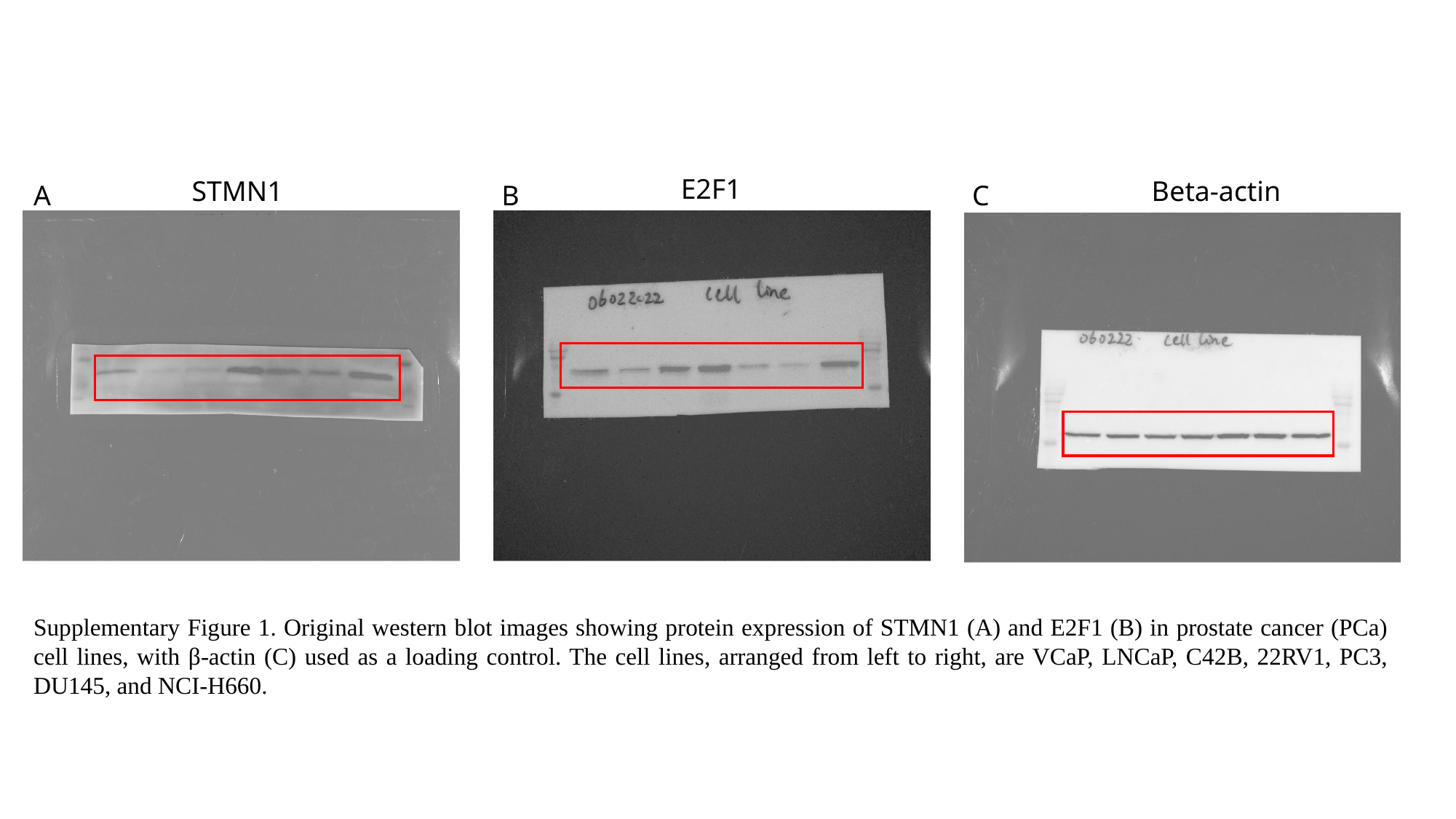

E2F1
STMN1
Beta-actin
A
B
C
Supplementary Figure 1. Original western blot images showing protein expression of STMN1 (A) and E2F1 (B) in prostate cancer (PCa) cell lines, with β-actin (C) used as a loading control. The cell lines, arranged from left to right, are VCaP, LNCaP, C42B, 22RV1, PC3, DU145, and NCI-H660.
